# Non-surgical treatment of lateral malleolar fractures is safe: long-term follow-up of a comprehensive treatment algorithm

**DOI:** 10.1186/s12891-024-07924-x

**Published:** 2024-10-16

**Authors:** Erik Börjesson, Karolina Johannesson, Jan Ekelund, Emilia Möller Rydberg

**Affiliations:** 1 Institute of Clinical Sciences, Sahlgrenska Academy, University of Gothenburg, Gothenburg, Sweden; 2 Department of Orthopaedics, Sahlgrenska University Hospital, Göteborgsvägen 31, Gothenburg, Mölndal, SE-431 80 Sweden; 3 Centre of Registers Gothenburg, Gothenburg, Sweden

**Keywords:** Ankle fracture, Swedish fracture register, Epidemiology, Fracture management, Treatment algorithm

## Abstract

**Introduction:**

A study at Sahlgrenska University Hospital (SU) found significant variation in the treatment of ankle fractures, leading to the development of a structured treatment algorithm (TA). The TA aimed to standardise treatment and reduce the number of unnecessary surgical procedures. A follow-up study concluded that the number of surgeries had significantly decreased since the TA was introduced. However, the long-term effects of the TA and the reduced number of surgical procedures remain unclear. The aim of the study was to analyse the long-term effects of a structured TA for ankle fractures, focusing on complications and reoperation rates.

**Method:**

The present study is a long-term follow-up of the same two cohorts of patients with lateral malleolar fractures of type AO/OTA44-B1, as studied in the previous studies on the TA for ankle fractures at SU. The current study compares a group of AO/OTA 44B1 fractures treated before introducing the TA, the pre-TA cohort (*n* = 410), with a group treated after introducing the TA, the post-TA cohort (*n* = 333). Both cohorts were followed for at least four years, and the outcomes were reoperation or complication. Reoperation was defined as any surgical procedure performed ≥ 30 days after the injury.

**Results:**

The results highlight a statistically significant reduction in the reoperation rate for lateral malleolar ankle fractures from 7.1 to 2.4% (*p* = 0.006) after introducing a TA that reduced the number of primary surgical procedures. Hardware-related issues were identified as the dominant cause of reoperation in both cohorts. Three major reoperations were observed in the pre-TA cohort compared to none in the post-TA cohort. The present study revealed no increase in the frequency of reoperations (late surgeries) due to non-union.

**Conclusion:**

The non-surgical treatment of stable ankle fractures does not lead to an increase in reoperations caused by non-union. A TA that reduces the need for primary surgical procedures for lateral malleolar fractures of type AO/OTA44-B1 has resulted in a significant decrease in reoperation rates and no increase in failure rates. This long-term follow-up demonstrates that a non-surgical approach to isolated lateral malleolar fractures is safe.

## Background

Ankle fractures are among the most common in the adult population and are the third most common fracture registered in the Swedish Fracture Register (SFR) [1, 2]. Ankle fractures can be the result of various types of trauma, but the most prevalent cause of ankle fractures is a fall at the same level, typically due to slipping, tripping, or stumbling [3]. An ankle fracture is a fracture to one or more of the malleoli, sometimes accompanied by injuries to the adjacent ligaments. Transsyndesmotic lateral malleolar ankle fractures (AO/OTA44-B) are the most common type, accounting for approximately 60% of all ankle fractures [3]. AO/OTA44-B fractures can be stable or unstable, depending on whether there is a fracture or ligament injury to the medial structures of the ankle. Isolated lateral malleolar fractures are classified as AO44-B1. An isolated trans-syndesmotic lateral malleolar fracture without signs of deltoid ligament injury can safely be treated without surgical intervention [5–8]. If combined with a ligamentous injury to the medial structures, the fracture is referred to as AO44-B2.1 and is no longer considered isolated [4]. To make informed decisions about treating ankle fractures, a thorough understanding of ankle biomechanics and anatomy is crucial for assessing ankle stability [9, 10].

The SFR is a national quality registry that collects information on different fracture types treated by orthopaedic surgeons in Sweden. Registrations performed by the treating physician include information about the cause of the injury, type of fracture, treatment provided, and subsequent outcomes [2]. The SFR classifies fractures using the AO/OTA 2007 classification system and considers fracture and ligament injuries [11]. The validity of the data in the register has been confirmed by multiple studies [12–14]. In 2015, a study was conducted at Sahlgrenska University Hospital (SU), revealing considerable variation in treating ankle fractures. This study demonstrated that accurately distinguishing between a stable, isolated lateral malleolar fracture and an unstable lateral malleolar fracture accompanied by medial ligament injury can be a complex and demanding task in daily clinical practice. The study also showed considerable variation within the same department regarding weight-bearing restrictions, treatment choice, and follow-up [15]. To address this issue, a structured treatment algorithm (TA) was developed to standardise the treatment of ankle fractures and reduce unnecessary surgical interventions. Before introducing the TA at SU, as in most other hospitals, no such TA had been in place for ankle fractures, which made the treatment decision primarily up to the surgeon’s preference. The introduction of the TA was aimed at enhancing the structure of treatment decision-making by providing clear guidance on treatment options and postoperative restrictions. A follow-up study conducted by Rydberg et al. in 2022 found that the TA significantly reduced the number of surgical treatments in patients with stable ankle fractures [16].

Despite these promising results, the long-term effects of reducing surgical treatment for lateral malleolar fractures remain unknown. Numerous patients are surgically treated despite having isolated, stable fractures suitable for non-surgical treatment. Therefore, the aim was to evaluate the long-term effects and safety of this TA for reoperations, complications, and delayed surgeries due to non-union.

## Methods

**Fig. 1:**
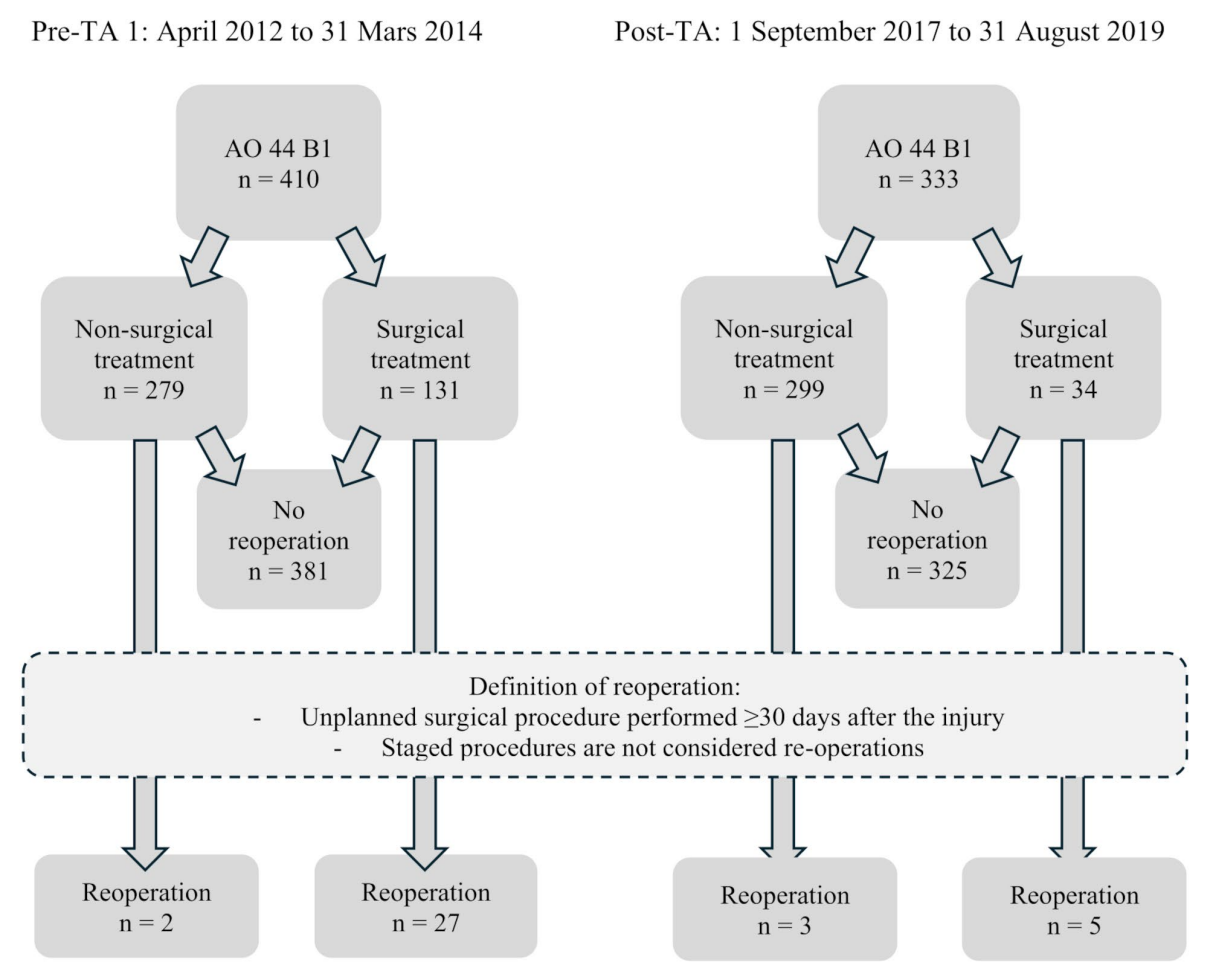
Flow chart illustrating the study cohorts before and after implementation of the treatment algorithm, includingpathways leading to reoperation

The present study is a long-term follow-up of the same two patient cohorts investigated by Rydberg et al. in 2022. Both cohorts include patients with lateral malleolar fractures registered as AO/OTA44-B1 in the SFR at the SU. The first cohort, the pre-TA cohort, comprises patients treated between 1 April 2012 and 31 March 2014 (*n* = 410) before introducing the TA with a mean follow-up time of 10.25 years. The second cohort includes patients treated after the TA’s introduction between 1 September 2017 and 31 August 2019 (*n* = 333) and has a mean follow-up time of 4.87 years. The follow-up period for both cohorts extended until 13 September 2023, at which point all patients had been followed for a minimum of four years (Fig. 1).

The study’s outcome was the occurrence of reoperation, which refers to an unplanned surgical procedure that takes place at least 30 days after the injury. This includes late surgeries attributed to non-union. The reoperations were categorised as major or minor. Major reoperations were defined as complications requiring surgical intervention (e.g., infection or failure) and minor reoperations were surgeries performed due to patient complaints (e.g., hardware removal). Planned surgical procedures, such as the removal of a syndesmotic screw, were considered as reoperations. Information on reoperations was extracted from the digital operating planning systems used at the SU. Additional examination of the medical records was undertaken to gain insight into the underlying factors behind each reoperation. The statistical software used was IBM SPSS Statistics version 29. To assess the time to reoperation, a comparison was made between the pre-TA and post-TA cohorts using Cox regressions, with cohort, sex, and age group as covariates. Continuous variables are presented as mean and standard deviation, while categorical data are presented as frequency and percentages. P-values < 0.05 were considered statistically significant.

## Results

There were 410 patients in the pre-TA cohort and 333 in the post-TA cohort, with a follow-up period of at least four years. Both cohorts contained more women (56.0%) than men (44.0%). The mean age at the time of injury was 52.1 years in the pre-TA cohort and 52.6 years in the post-TA cohort. Women had a higher mean age at the time of injury than men (54.5 years vs. 49.7 years (Table 1).

**Table 1:** Demographic data of patients with ankle fractures before (pre-TA) and after (post-TA) the introduction of a structured TA*

	Total *n* = 743	Pre-TA *n* = 410	Post-TA *n* = 333
Sex			
Male, n (%)	327 (44.0)	189 (46.1)	138 (41.4)
Female, n (%)	416 (56.0)	221 (53.9)	195 (58.6)
Mean injury age			
Total years (SD)	52.3 (19.2)	52.1 (19.2)	52.6 (19.4)
Male years (SD)	49.7 (19.8)	48.2 (19.5)	51.6 (20.1)
Female years (SD)	54.5 (18.5)	55.5 (18.3)	53.3 (18.8)
Age groups			
16–39 years, n (%)	203 (27.3)	113 (27.6)	90 (27.0)
40–59 years, n (%)	259 (34.9)	141 (34.4)	118 (35.4)
60–99 years, n (%)	281 (37.8)	156 (38.0)	125 (37.5)

In the pre-TA cohort, 161 (39.3%) patients had positive clinical findings indicating medial ligament injury, while 167 (40.8%) did not. In the post-TA cohort, 95 (28.5%) patients had clinical indications suggestive of medial ligament injury, and 193 (58.0%) did not manifest any such findings.

### Reoperations

In the pre-TA cohort, 29 of 410 patients (7.1%) underwent reoperation. In the post-TA cohort, 8 of 333 patients (2.4%) required reoperation (Table 2).

In the pre-TA cohort, 131 patients (31.9%) were initially treated surgically, and 27 (20.6%) later required a reoperation. In the post-TA cohort, 34 patients (11.3%) underwent primary surgical treatment, and 5 (14.7%) later needed a reoperation.

In the pre-TA cohort, 279 patients received non-surgical treatment initially, with only 2 cases (0.8%) requiring a subsequent reoperation. Among the patients in the post-TA cohort, 299 patients underwent non-surgical treatment initially, with subsequent reoperation necessary for 3 patients (1.0%).

**Table 2:** Descriptive analysis of the number of reoperations based on the initial treatment method

		Total, *n*	No reoperation, *n* (%)	Reoperation, *n* (%)
Non-surgical treatment				
	Pre-TA	279	277 (99.3)	2 (0.7)
	Post-TA	299	296 (99.0)	3 (1.0)
	Total	578	573 (99.1)	5 (0.9)
Surgical treatment				
	Pre-TA	131	104 (79.4)	27 (20.6)
	Post-TA	34	29 (85.3)	5 (14.7)
	Total	165	133 (80.6)	32 (19.4)
Total				
	Pre-TA	410	381 (92.9)	29 (7.1)
	Post-TA	333	325 (97.6)	8 (2.4)
	Total	743	706 (95.0)	37 (5.0)

**Fig. 2:**
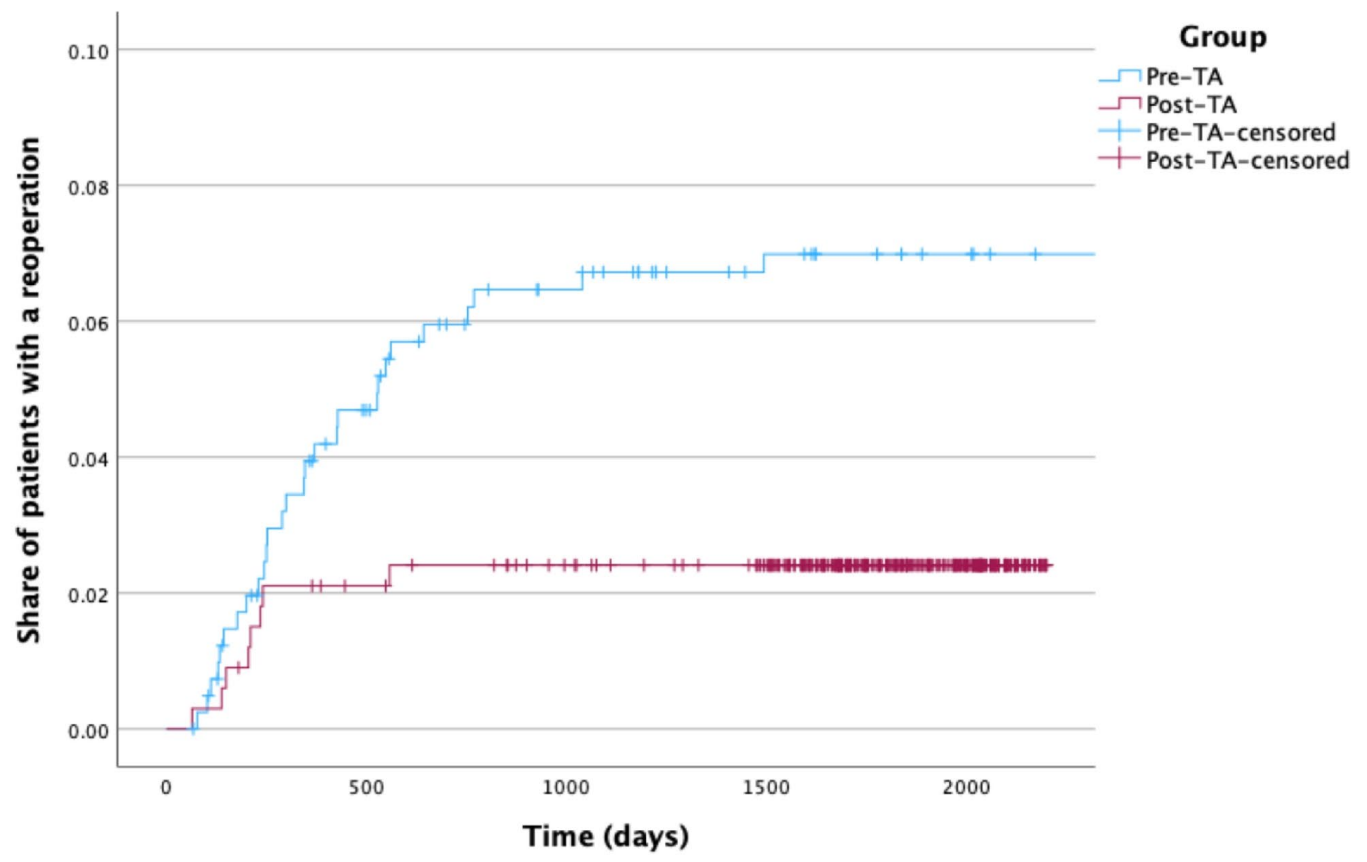
Kaplan-Meier plot of time to reoperation

A statistically significant difference was observed in the number of reoperations between the pre-TA (20.6%) and post-TA (14.7%) cohorts (hazard ratio, HR = 0.33; *p* = 0.006). Figure 2 depicts a Kaplan-Meier plot of the time to reoperation. An event is defined as reoperation (Table 3; Fig. 2).

**Table 3:** Estimated hazard ratio (HR) from Cox regression for time to reoperation using sex and age group as covariates

	HR	95.0% CI		*P*-value
		Lower	Upper	
Cohort Pre-TA REF	0.330	0.150	0.725	0.006
Sex male REF	1.397	0.714	2.732	0.329
Age group 16–39				REF
Age group 40–59	0.916	0.457	1.838	0.805
Age group 60–99	0.183	0.060	0.555	0.003

Three major reoperations in three patients were performed, all documented in the pre-TA cohort. In two of these patients, an evaluation of radiographic images taken at the time of the injury showed a medial clear space of 14.5 mm and 9 mm, indicating an incorrect classification of the fracture as AO/OTA-B1 in the SFR. The third patient underwent a major reoperation because of a severe infection at the surgical site following primary surgery. The patient underwent antibiotic treatment and was subsequently hospitalized for the removal of the plate and screws. No major reoperations were observed in the post-TA cohort.

## Discussion

The major finding of this long-term follow-up study is that treating stable ankle fractures non-surgically is safe and does not lead to an increased risk of non-union, mal-union, or increased reoperation rates. Based on a minimum of four years of follow-up, the current study indicates that there was no increase in the rate of reoperations; in fact, it significantly decreased after introducing the TA. This study also shows no increase in the frequency of late surgeries due to non-union. Our results provide insight into the effectiveness and safety of implementing a standardised TA for lateral malleolar fractures.

The present study expands on Rydberg et al.’s ., showing that implementing a standardised treatment approach for lateral malleolar fractures led to a notable reduction in surgical intervention [17]. Our study corroborates these findings and concludes that the non-surgical management of stable ankle fractures is a safe and reliable long-term option.

Reoperation was defined in the present study as an unplanned surgical treatment occurring ≥ 30 days post-injury, regardless of the initial procedure (surgical or non-surgical). Planned procedures, such as the removal of a syndesmotic screw, were not considered reoperations. As planned secondary procedures were not classified as a reoperation, the higher number of reoperations found in the pre-TA cohort cannot be explained by more patients receiving primary surgical treatment before introducing the TA.

Most reoperations in both cohorts were minor reoperations. While minor reoperations are typically straightforward and routine procedures, they often require general anaesthesia and take place in an operating theatre. Reith et al. surveyed patients with hardware-related pain. These authors found that the most frequent indications for metal implant removal in the ankle were pain, personal preference, professional recommendation, or function impairment. After hardware removal, 52% of their patients reported pain relief, while 52% experienced improved functionality [18].

All surgical procedures carry a risk of complications, including infections. Andersen et al. [19] concluded that there was a 5% risk of wound infections after syndesmotic screw removal. By minimising unnecessary surgical interventions for ankle fractures, including primary and planned secondary procedures such as syndesmotic screw removal, the potential for complications can be reduced, resulting in enhanced patient satisfaction.

This study identified three major reoperations, all occurring within the pre-TA cohort. Two of the patients had an increased medial clear space, indicating the presence of a medial ligament injury. AO/OTA44-B1 should not have any significant medial ligament injuries, and the correct classification for these fractures should be AO/OTA44-B2.1, for which primary surgical treatment was recommended. It can be deduced that these ankle fractures were incorrectly classified at the group level in the AO/OTA classification within the SFR. Juto et al. concluded that the SFR classification of ankle fractures is reliable. However, they also acknowledged that classifying fractures at the group level can pose challenges [12]. The Juto et al. study evaluated the precision of ankle fracture classifications in the SFR by applying stringent AO/OTA classification guidelines on random samples. The results showed an accuracy of 88% for the AO/OTA type. However, the group-level accuracy was slightly lower at 74%. Consequently, distinguishing the various subgroups within type B injuries becomes more difficult. This observation aligns with the findings of the current study, wherein the two significant reoperations likely resulted from misclassified fractures and subsequent incorrect treatment methods.

The third major reoperation was correctly classified in the SFR as an AO/OTA44-B1 fracture. A deep infection following the initial surgery led to the need for this reoperation. The patient required hospitalisation for i.v. of antibiotics, and the medical team decided to remove the plate and screws to eradicate the infection. Serious infections are not uncommon after surgery. For instance, Cammas et al. identified infection as the second most common complication [20]. A retrospective observational study at SU by Bergström et al. investigated the incidence of surgical site infections (SSIs) for surgically treated ankle fractures [21]. The study, including 480 patients who underwent primary surgery, revealed that 10.2% developed an SSI. In the study, patients who underwent reoperation due to infection usually had one to four reoperations before the infection resolved. Moreover, the infected patients required additional medical follow-ups and extended hospital stays. Hence, surgery carries significant risks, and the most effective approach to mitigating these risks is by avoiding unnecessary surgeries.

Five of our patients required surgical intervention at a later stage due to non-union. The mean age of injury for these patients was 45 years, which is younger than the overall mean age in the study. This finding is consistent with existing literature, which suggests that non-union is more common among younger patients. According to Mills et al., the highest risk of non-union occurs in individuals aged 25–44 years [22]. The primary evaluation conducted at the Accident & Emergency Department accurately indicated no surgical need for the five non-unions in this study. Consequently, the patients were treated with orthosis, in accordance with the TA recommendation. Nevertheless, the fractures failed to heal adequately, prompting the patients to seek medical attention due to persistent pain and discomfort. A radiographic examination confirmed that the fractures had not healed properly and required surgical intervention. It was not possible to identify a discernible explanation for the unsuccessful healing in all five patients. Non-union is a recognised orthopaedic problem. Mills et al. suggested that 1% of all ankle fractures result in non-union [22]. This suggestion is consistent with the present findings, where the total non-union risk was 0.9%, with 0.8% in the pre-TA cohort and 1.1% in the post-TA cohort. Thus, the risk of non-union is the same regardless of the TA, suggesting no elevated risk of non-union with fewer primary surgeries.

The study’s main strengths lie in the cohort size, which included 743 patients with lateral malleolar ankle fractures and the minimum four-year follow-up for all patients. Additionally, the thorough review of medical records for all reoperations facilitated a deeper understanding of the root causes, contributing to the study’s overall strength.

One limitation of the study is that it only includes reoperations performed at SU in patients who sought medical care voluntarily. Therefore, the study does not include patients who might have undergone reoperations at other facilities in Sweden or abroad. This study excludes complications that did not require surgical intervention. Even though this study offers insights into the benefits of implementing a structured treatment approach in reducing long-term complications and reoperations for AO/OTA44-B1 fractures, there is a lack of data on the well-being of patients who did not seek medical care beyond the initial follow-up period. A recent study that included 11 751 patients > 18 years who suffered an ankle fracture and who had completed PROM evaluation at day 0 and 1 year after the trauma showed that the classification of ankle fractures according to AO/OTA 44A1-C3 was prognostic with more complex fractures associated with poorer PROM. A patient-reported outcome measurement (PROM) would provide valuable additional information to evaluate the TA’s long-term outcome [24] fully.

Future studies in this field should investigate how an ankle fracture affects patient-reported outcome measures (PROM), pain management, and return to work.

## Conclusions

This study provides evidence that non-surgical treatment of stable ankle fractures does not result in a higher rate of reoperations or late surgeries caused by non-union. In fact, a structured TA that reduces the number of primary surgical procedures for lateral malleolar fractures of type AO/OTA44-B1 also led to a significant reduction in reoperation rates and no increase in failure rates. The results of this extended follow-up indicate that a non-surgical treatment method for isolated lateral malleolar fractures is deemed safe.

## Data Availability

The datasets used and analysed during the current study are available from the corresponding author upon reasonable request.

## Abbreviations

SU: Sahlgrenska University Hospital
TA: Treatment algorithm
AO: Arbeitsgemeinschaft für Osteosynthesefragen
OTA: Orthopaedic Trauma Association
SFR: Swedish Fracture Register
MRI: Magnetic resonance imaging
